# Graphite Furnace AAS: Application of Reduced Palladium as a Chemical Modifier

**DOI:** 10.6028/jres.093.115

**Published:** 1988-06-01

**Authors:** D. E. Shrader, L. M. Beach, T. M. Rettberg

**Affiliations:** Varian Instrument Group-AA Resource Center, 205 W. Touhy Ave., Park Ridge, IL 60068

Chemical modification techniques are widely used in graphite furnace atomic absorption spectrometry (GFAAS). Palladium is a very effective chemical modifier and can be used to stabilize many elements to several hundred degrees higher than the temperatures possible with current methods [1–9]. Of the elements tested, the greatest temperature shifts are achieved for the semi-metallic elements such as As, Se, Te, Bi, Sb, Pb, Tl, Ga, Ge and P. Ash temperatures can be raised 400–800 °C higher than current methods allow. Temperature shifts are somewhat less for the transition elements and ash temperatures can be raised 200–500 °C. Palladium has no similar effect on elements in Groups I and II of the Periodic Table. The change in stability is believed to be due to the formation of an intermetallic species. This improvement in stability permits more efficient removal of matrix constituents during the ash step and vaporization into a hotter environment during the atomize step. Background and interference problems are thus reduced or eliminated.

Steps taken to guarantee that palladium is present as the reduced metal as early as possible greatly improve performance of the modifier. The palladium modifier solution can be pre-injected and the graphite tube heated to 1000 °C. Such a method has been used to stabilize mercury [10]. It is assumed that at this temperature palladium metal is present on the graphite surface. The sample can then he introduced. The addition of a reducing agent such as 5% hydrogen in 95% argon, ascorbic acid, or hydroxylamine hydrochloride also appears to guarantee that the palladium is present as the metal early in the temperature program. The use of hydrogen as a reducing agent appears to be the method of choice for a number of reasons. It is cleaner, leaves no residue, and is less subject to contamination. It is also easy to use. A pre-mixed gas of 5% hydrogen in 95% argon can simply be introduced into the furnace. More importantly, the problem encountered with high concentrations of nitric acid is eliminated with the use of hydrogen.

Reduced palladium metal allows the retention of the analyte element on the graphite surface until a higher gas phase temperature is achieved. This appears to give many of the analytical advantages normally associated with platform atomization while using the simpler wall atomization technique.

Investigations to elucidate the mechanism of palladium chemical modification have been conducted. Also, comparison of palladium modifier methods with current modifier methods in spike recovery studies from difficult matrices was accomplished.

Scanning electron micrographs of graphite surfaces with palladium deposits obtained by different reduction methods were obtained to investigate whether the physical form of palladium influenced the modifier behavior. It was found that reduced palladium metal was indeed present on the graphite surface after reduction and that the most effective reduction method was the use of 5% hydrogen in 95% argon with a palladium solution containing 1% glycerol. The scanning electron micrograph of the surface produced under these conditions is shown in figure 1. The palladium particles are considerably smaller and more highly dispersed than those produced by other methods. The average particle diameter is 0.05–0.15 μm. The smaller particles result in a great increase in palladium surface area. Spike recovery studies from difficult matrices showed that smaller, highly dispersed particles produced improved interference performance.

The interference performance of a palladium/hydrogen/glycerol method for tin was compared with the performance of a commonly used method. The commonly used method requires the use of the platform and chemical modifiers of ammonium dihydrogen phosphate and magnesium nitrate. Wall atomization was used for the palladium method. The results of this study are listed in table 1. While both methods performed well in concentrated HCl, in most instances the palladium gave slightly better interference performance. It was significantly better in overcoming interferences from NaCl and seawater matrices. Other elements have been tested in the same matrices and, in virtually every instance, palladium gave as good as or better interference performance (wall) as did the currently used methods (platform).

The palladium modifier method has been utilized with numerous real samples as well as synthetic interferent matrices to test its applicability. The direct determination of selenium in biological fluids has shown excellent accuracy using palladium modification. Thallium has been accurately determined in brackish waters, soil digests and digested fish tissue. Work with other real samples is continuing.

In many situations, quite low concentrations of palladium may be used and accurate results still obtained. Table 2 compares recommended chemical modifier concentration ranges. The use of lower modifier concentrations decreases the possibility of analyte contamination and associated analysis problems.

In summary, reduced palladium chemical modification for graphite furnace AAS has been shown to be very useful. It can be used to improve difficult graphite furnace analyses. It has been applied to over 20 analyte elements and in each case has produced beneficial effects. It does not require the added complexity of platforms as it produces the same effects chemically. Reduced palladium may be as close to a “universal” modifier as possible for graphite furnace AAS.

## Figures and Tables

**Figure 1:**
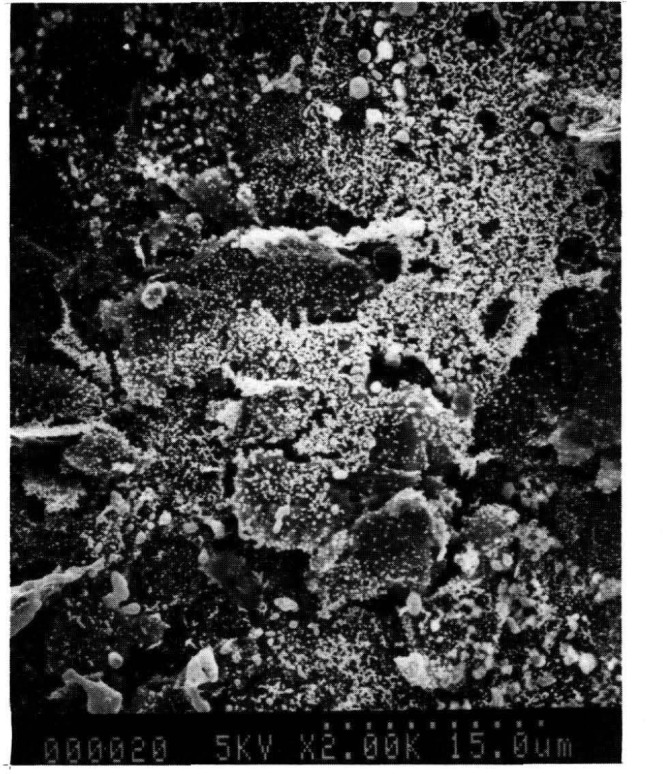
Scanning electron micrograph of reduced palladium metal on graphite surface (20,000× magnification).

**Table 1:** Tin recoveries from interferent matrices

Interferent	Current method 200 *μ*g NH_4_H_2_PO_4_ 10 *μ*g Mg(NO_3_)_2_ Platform	Palladium method 20 *μ*g Pd 150 *μ*g glycerol 5% hydrogen Wall
5 *μ*L 2.5% NaCl (125 *μg*)	22%^a^	91%^a^
	26%^b^	110%^b^
5 *μ*L 5.0% NaCl (250 *μg*)	0	92%
	0	107%
5 *μ*L Seawater	0	87%
	0	99%
5 *μ*L concentrated HCl	97%	96%
	92%	100%
5 *μ*L concentrated HNO_3_	79%	92%
	87%	93%
5 *μ*L 20% H_2_SO_4_	39%	60%
	58%	79%
5 *μ*L 0.5% Na_2_SO_4_ (25 *μ*g)	87%	101%
	85%	101%
5 *μ*L 1.0% Na_2_SO_4_ (50 *μ*g)	82%	103%
	85%	100%

a Peak height.

b Peak area.

**Table 2:** Recommended modifier concentration ranges (*μ*g/mL)

Modifier	Element	Current methods	Palladium method
Nickel	As	Up to 5000	50–1000
	Se	Up to 5000	50–1000
Mixed	Cd	Up to 20,000[1000]	50–1000
PO_4_ + [Mg(NO_3_)_2_]	Pb	Up to 20,000[1000]	50–1000
	Sn	Up to 20,000[1000]	50–1000
Mg(NO_3_)_2_	Fe	Up to 5000	50–1000
	Mn	Up to 5000	50–1000
